# Intimate partner violence types and symptoms of common mental disorders in a rural community of Chiapas, Mexico: Implications for global mental-health practice

**DOI:** 10.1371/journal.pone.0256850

**Published:** 2021-09-02

**Authors:** Mercedes Aguerrebere, Sonia M. Frías, Mary C. Smith Fawzi, Rocío López, Giuseppe Raviola

**Affiliations:** 1 Compañeros En Salud México A.C, Angel Albino Corzo, Chiapas, Mexico; 2 Department of Global Health and Social Medicine. Harvard Medical School, Boston, Massachusetts, United States of America; 3 Centro Regional de Investigaciones Multidisciplinarias, Universidad Nacional Autónoma de México, Cuernavaca, Morelos, Mexico; 4 Escuela de Medicina y Ciencias de la Salud TecSalud, Instituto Tecnológico de Monterrey, Monterrey, Nuevo León, Mexico; 5 Massachusetts General Hospital, Boston, Massachusetts, United States of America; 6 Partners in Health, Boston, Massachusetts, United States of America; Universidad Pública de Navarra, SPAIN

## Abstract

This paper examines the scope and characteristics of male-to-female intimate partner violence in southern rural Chiapas, Mexico, and its association with depression and anxiety symptoms, highlighting the role of partner controlling behaviors. Participants were selected by random sampling. One-hundred and forty-one women >15 years participated in the study. Data was obtained through an adapted version of the National Survey of the Dynamics of Household Relationships (ENDIREH) intimate partner violence scale, the Patient Health Questionnaire-9 for depression symptoms and the Generalized Anxiety Disorder-7 for anxiety symptoms. Quantitative results indicated a 66.4% lifetime prevalence of physical and/or sexual IPV among ever-partnered women 15 years or older (95% CI: 57.5–74.5%). Forty percent (95% CI: 32.0–49.7%) of them reported having experienced physical and/or sexual violence with high partner control (HC-IPV), and 25.8% (95% CI: 18.5–34.3%) reported having experienced physical and/or sexual violence with low or moderate partner control (MC-IPV). Lifetime experience of HC-IPV was significantly associated with moderate-severe depression symptoms (RR = 5.8) and suicidality (RR = 2.08). While partner alcohol abuse was associated with a 3.06 times higher risk of lifetime physical and/or sexual IPV, 30.9% of women mentioned that their partners were never drunk when violence occurred. Interestingly, high partner alcohol abuse was more frequent among women who reported HC-IPV compared to MC-IPV. Implications for global mental health practice are discussed.

## Introduction

International evidence shows that intimate partner violence (IPV) increases the risk of mental disorders [1]. Different contexts—influenced by socioeconomic status, race/ethnicity, marginalization and access to services—may influence how women experience IPV and the resources available to cope with it. Likewise, different IPV dynamics, such as those that entail partner’s controlling behaviors (CB), may have distinct effects on the mental health of victims and service providers may require different approaches to care. Sociologist Michael Johnson [2] uses the term intimate terrorism (IT) to describe the violence that a partner (a man 90% of the times) uses as a mean to control the victim. Control is defined as the attempt to dominate one’s partner and the attempt to have absolute power in the relationship by including several tactics such as “economic abuse, emotional abuse, the use of children, threats and intimidation, invocation of male privilege, constant monitoring, blaming the victim, threats to report to immigration authorities or threats to out a person to work or family” [3 p. 290]. In contrast, the term situational couple violence (SCV) refers to violence in the context of conflict, without the intention control one’s partner. The CB that characterize IT [2] are usually classified as economic or emotional violence in IPV scales, which may be inaccurately perceived by service providers and researchers as less severe than physical and sexual violence. Consequently, the later types of IPV are often the ones addressed by health, social and legal services providers, disregarding the role that partner control has on women’s mental health, liberty, and autonomy [4].

Likewise, most research “takes violent acts as its unit of analysis” [5 p. 202], dismissing the negative cumulative effect that contextual factors—including control tactics, concern for children’s safety and availability of services—have on an individual’s wellbeing [5, 6] There have been few studies addressing the differential impact that CB in violent dynamics might have on women’s mental health [6–8]. These studies have shown that IPV with CB (IT) increases over 2.5 times the risk of mental health problems such as depression, anxiety attacks, and lower self-esteem compared with IPV without CB (SCV) [6, 7]. Likewise, IT has been found to be associated with higher posttraumatic stress symptoms and more frequent use of painkillers and tranquilizers [8].

In addition to CB, traditional gender norms—social norms on gender stereotypes and roles that individuals are expected to comply with according to their sex [9]—also exert control on women, influence their experiences of IPV, and impact their mental health. In rural Chiapas, gender norms dictate that women should stay in the household doing housework and caring for children and family, and not wander around nor go out by themselves. These gender norms limit women’s access to financial resources, promote social isolation and constrict women’s social networks, leaving them with little support to cope with mental health problems as well as leave a violent relationship [10–14]. In Mexico, being dedicated almost exclusively to housework, caring for family members, and experiencing gender-based violence have been strongly associated with depression [15]

The context of gender inequality could also play a role in the link between partner alcohol abuse and IPV. Existing research suggests that the same gender norms could endorse both, alcohol abuse among men and men’s CB against women [16, 17] Moreover, because alcohol hampers control of impulsive behavior, and alters judgement of perceived social threats [17], it could trigger IPV in the context minor conflict if the perpetrator feels the need to exert control over his partner. In addition, these norms can accept a “time out behavior” during which violence perpetrated by men under the influence of alcohol against women is condoned [16].

Over the last decade there has been extensive epidemiologic research on violence against women in Mexico [18–23]. However, to our knowledge, there is no research that has examined the experience of IPV with and without CB and its association with women’s mental health, in rural Mexico. The aim of this study was to evaluate the different scope and characteristics of male-to-female intimate partner violence, with or without high CB, and its association with depression and/or anxiety symptoms and suicidality among women in a non-indigenous rural community between the Sierra and Fraylesca regions of Chiapas. Chiapas has the lowest ranking on structural gender equity among the 32 Mexican States [24]. In this setting, structural gender inequality, poverty and the lack of health and social services intersect with the experience of IPV, resulting in chronic suffering among women [25]. Because “coercion in the context of structural inequality has different dynamics and individual and societal consequences” [5, p. 202], it is important to study IPV with and without CB and its association with women’s mental health in this specific setting.

## Methods

### Ethical considerations

The study design followed the WHO “Ethic and Safety Recommendations for conducting research on Domestic Violence Against Women” [26]. The research study received ethical approval from the Institutional Review Board of the Harvard Medical School Office of Human Research Administration and the Chiapas Health Institute. Oral consent was obtained.

### Study setting

Chiapas is Mexico’s poorest state, with 51% of the population living in rural areas, 76.2% of the population living in poverty (measured as having insufficient income for basic goods and services and at least one of the following characteristics: a) educational lag; b) poor living standard–inadequate construction material of the dwelling, number of people per room–; c) insufficient or absence of access to household basic services; d) lack of access to health services; e) lack of security, and; f) lack of access to quality and nutritious food), 31.8% living in extreme poverty (insufficient income for adequate nutrition plus lacking three or more of the above) [27, 28], and almost half of children under five years old suffering from chronic malnutrition [29]. This situation has deep historical roots: since the 1800s, the region’s economy has been subjected to the international market value of coffee [30]; in the early 1900s, gender norms facilitated exclusive land tenure by men during agrarian reforms [31] when communities organized in an *ejidal* structure (communal arable land in which users have usufruct rather than ownership). Even though in the 1980s and 1990s social movements gave rise to coffee cooperatives, families still depend on the value of coffee in the stock market today, and it is rare for women to acquire land tenure [32].

Deep poverty is accompanied by a small number of mental health providers in Chiapas. With a rate of 0.57 psychiatrists per 100,000 inhabitants in the state, 30 psychiatrists serve a population of over five million people [33]. Moreover, only four psychiatrists and 210 psychologists work for the public health sector [34], which serves 82% of Chiapas’ population [35]. These providers are centralized in cities located four to ten hours away from the study setting. Likewise, services for women who are victims of IPV are scarce and often insufficient because they are unable to comprehensively fulfill their needs. Social workers, lawyers and mental health professionals are not available in this setting. and the few shelters for women experiencing IPV in the state are more than three hours away. Nationally, only 2 out of 10 female victims of IPV seek government assistance, with rural women being less likely to seek help than their urban counterparts in governmental agencies [20].

It is within this context that *Compañeros En Salud México A*.*C* (CES)—a non-profit organization that partnered with the Ministry of Health of Chiapas to provide primary health care in the Sierra and Fraylesca regions—strives to provide quality mental health services. It is also in this context that this study takes place.

### Study design, sampling and recruitment

The study was a cross-sectional survey conducted between July and August 2017, in a highly marginalized rural community of about 1200 inhabitants in Chiapas, Mexico. Inclusion criteria included being female, at least 15 years old, and a current resident of the community. Exclusion criteria included a) hearing, language or cognitive impairments (*n* = 2, 1.42%; data was collected using face to face interviews), or b) having another member of the household participating in the study (*n* = 7, 4.96%; to ensure confidentiality). To select participants a comprehensive list of households with women 15 years and older was obtained from the staff at the CES–operated health clinic. Households on the list were numbered and numbers were randomly sequenced for top-down selection. Eligible women within each selected household were likewise numbered and randomly selected. A total of 159 eligible women were selected. When a selected woman had moved, died, or did not fulfill participation criteria, the household with the next number in the random sequence list was selected. When a selected woman had moved to a different household within the community (usually due to marriage/cohabitation with partner), and a woman on that new household had already been selected, that participant was excluded, and the household with the next number in the random sequence list was elected. In the end 289 households were screened to reach a target of 159 eligible women. Of those 159 women, 18 did not participate in an interview after rescheduling twice; 141 women participated in the study (89% response rate) of which 128 had ever lived with a partner.

### Quantitative data collection and measurements

The survey interviews were conducted face-to-face by the first author and three female trained research assistants using tablets and Commcare® software. Interviews were conducted in a private room where the conversation could not be overheard. Tablets were password protected and kept at a secure location. Data were removed from the tablets after completion of data collection.

#### Sociodemographic data

Age, literacy (knows how to read and write), years of schooling and educational attainment, age at first cohabitation, number of children, average age of children in the household, family income, participation in governmental conditional-cash-transfer program *Prospera* (CCT) and food insecurity were recorded. Women’s access to economic resources such as land ownership and self-employment, and hours spent in housework were also assessed. Finally, food security was measured following Semali et al.’s [36] methodology in other low-resource rural areas. Age at first cohabitation with a partner, number of children (agency on sexual and reproductive rights, use of contraception), women’s employment (rare in the study setting due to rigid gender roles), coffee crop ownership (ability to fulfill the provider role), and controlling behaviors (submission of women to male authority) are linked to traditional gender beliefs and norms and previous literature has shown their association with mental health [37].

#### IPV related variables

Physical and sexual IPV, as well as lifetime partner CB, were measured using a locally adapted version of the 2016 National Survey of the Dynamics of Household Relationships (*ENDIREH*), a nationally representative household survey that measures violence against women [38]. Cronbach’s alpha resulted in 0.80 for the physical violence scale, 0.79 for the sexual violence scale and 0.86 for the CB scale. Analysis was done with the subset of women who had ever lived with a partner (*n* = 128).

Frequency of physical, sexual and controlling violent events was assessed as follows: never happened, happened once, happened a few times, happened many times. These categories were provided in the survey and are contingent upon each respondent’s consideration.

Based on previous research [39] severity of IPV (physical and sexual) was categorized as low, moderate or severe. These categories were created through a combination of the frequency of violent events and the severity of events: being kicked, tied-up, choked, attacked with a machete/knife and attacked with a gun where considered severe regardless of frequency; being pushed, pulled by the hair, slapped, hit, or have objects thrown at her where defined as follows: a) low severity when the event(s) occurred once, b) moderate severity when events happened a few times, and c) high severity when events happened many times. Similarly, being coerced to have sex, or forced to do specific sexual activities while having sex were categorized as follows: a) low severity if it happened once, b) moderate severity if it happened a few times, and c) severe if it happened many times. In the case of having been forced to have sex through physical force, it was considered a) moderate severity when it happened once, and b) severe when it happened more than once. IPV was labeled as low, moderate or severe based on the physical or sexual violence item that was labeled most severe for each woman.

Fourteen control tactics were asked to measure CB: 1) not permitting her to work outside of home; 2) taking away her property, belongings or money; 3) not permitting her to visit her family; 4) not providing with money for basic household needs when he did have; 5) humiliating, shaming or insulting her; 6) falsely accusing her of being unfaithful; 7) frightening her or making her feel afraid of him; 8) locking her in; 9) spying or monitoring her; 10) threatening her with throwing her out of the house; 11) putting the children against her; 12) threatening her with taking the children away from her; 13) threatening her with abandoning her; 14) threatening her with killing her. Partner CB level was categorized as: a) high with more than four control tactics were reported (highest tercile), b) moderate with one to four control tactics were reported, and c) no control when no control tactics reported. High-control IPV (HC-IPV) and moderate-control IPV (MC-IPV) categories were created to reflect Johnson’s suggested categories of Intimate Terrorism and Situational Couple Violence, respectively [2]. IPV was labeled as HC-IPV whenever a) control was high irrespective of the severity of physical or sexual violence, and b) control was moderate and severity of physical or sexual violence was high. IPV was labeled as MC-IPV when a) severity was low, and control was none or moderate, b) when severity was moderate and control none or moderate, and c) when physical or sexual violence was severe and no control tactics were reported.

#### Mental health

The Patient Health Questionnaire-2 (PHQ-2) was used to screen for depression [40]. If a response was positive for either of the PHQ-2 questions, the PHQ-9 questionnaire for depression was administered [41]. The PHQ-2 and PHQ-9 questionnaires have been previously validated for screening, probable diagnosis of depression in this specific population (Cronbach’s alpha of 0.81, and good predictive validity) [42]. Suicidality was measured with the ninth question of the previously validated PHQ-9 version “over the last two weeks, have you thought you would be better off dead?”. Anxiety symptoms were measured using the Generalized Anxiety Disorder -7 (GAD-7) questionnaire [43]. In our study sample, PHQ-9 and GAD-7 had a Cronbach’s alpha of 0.86 and 0.78, respectively.

Partner alcohol abuse was assessed by asking women the frequency in which her partner got drunk: never, less than once a month (low), at least once a month but less than every week (moderate), at least once every week (high).

### Data analysis

We first conducted a descriptive analysis of socio-demographic data. Second, lifetime and one-year prevalence of IPV were calculated with point estimates and 95% confidence intervals, both aggregated and stratified by type (physical, sexual). Lifetime prevalence of partner CB was likewise calculated. Prevalence ratio estimates for different types of violence (IPV, MC-IPV, HC-IPV) were calculated for each socio-demographic characteristic. To examine associations between demographic data and different types of violence, Chi-square or Fisher’s exact tests were used for binary and categorical variables; Wilcoxon rank sum tests was used for non-parametric continuous variables. Likewise, Chi-square or Fisher’s exact tests were used to measure the association between IPV, MC-IPV, and HC-IPV, with depression and/or anxiety symptoms, and partner alcohol abuse. To calculate relative risks, each constructed category of IPV was compared with the reference category (no IPV). To facilitate understanding of results prevalence ratios are reported as risk ratios. Logistic regression was used to examine associations between ordinal variables as well as for multivariate analysis to examine for potential confounders identified *a priori*.

Depression and anxiety symptoms were categorized as mild symptoms if the PHQ-9 or GAD-7 score, respectively, was greater or equal than five and less than 10, and moderate-severe symptoms of either score was equal or greater than 10. To calculate relative risks, mild symptoms were compared to zero-minimal symptoms (PHQ-9 or GAD-7 score <5), and moderate-severe symptoms (PHQ-9 or GAD-7 ≥ 10) were compared to zero-minimal symptoms and mild symptoms (PHQ-9 or GAD-7 ranging from 0 to 9). Those in the moderate-severe category were considered to have symptom levels comparable with major depressive or anxiety disorder. Having a common mental disorder (CMD) was defined as having moderate-severe symptoms of either depression or anxiety or both [42–44]. This definition of CMD was used when examining the associations with types of IPV.

## Results

### Sociodemographic data

Table 1 shows the demographic and socioeconomic characteristics of the study population. Of 141 women surveyed, 77.3% knew how to read and write, and 53.9% had completed elementary school or more. The median age of participants was 32 (15–85) years, the median age at first cohabitation was 18 (16–21), and the median number of children participants had had was 4 (1–18). Of the 128 women who had lived with a partner (91%), 111 were currently cohabitating (79%). Among cohabitating women 56.3% live in their partner or in-laws house, and 26.6% reported they owned the house, either exclusively (11.7%) or along with her partner (14.8%). The remaining women reported their parents or other relatives owned the house. In terms of economic characteristics, 92.7% of women’s partners worked in coffee-crops of whom 8.8% do not have coffee crops of their own. Last year’s average annual income from coffee was 11,750 MXN (4,600–19,000 MXN), equivalent to 3.7 USD purchasing power parity (PPP) per family per day. Of all women surveyed 22% reported having spare cash available in case of need, 12.1% owned land, and 21% had a small business. Among those with small businesses, only 16.7% (*n* = 5) reported having spare cash available in case of need. More than half of women surveyed reported that they did not have access to any financial resource. Only 69.5% of the interviewees receive support from *Prospera*, and only 44% of the participant’s families receive cash from other governmental programs such as *Procampo* (for agricultural development) and *Conafor* (to assure environmental protection). Finally, close to one in three families faced food insecurity during the 12-monts prior to the survey completion.

**Table 1:** Demographic and socioeconomic characteristics (*n* = 141).

Demographic	Median (min-max) / *n* (%)	
Age	32 (15–85)	
Literacy (read and write)	109 (77.3%)	
Years of education completed	6 (0–15)	
Level of education		
None	33 (23.4%)	
Primary school	32 (22.7%)	
Middle school	36 (25.5%)	
High school	7 (4.9%)	
College	1 (0.7%)	
Marital status		
Single	13 (9.2%)	
Civil marriage	3 (2.1%)	
Religious marriage	24 (17.0%)	
Cohabitating	81 (57.5%)	
Separated/divorced	7 (5.0%)	
Widowed	11 (7.8%)	
Age at first cohabitation^a^	18.69 (12–33)	
First cohabitation before 17 years	39 (31.2%)	
Have children	91 (90.8%)	
Number of children	4 (1–18)	
**Socioeconomic**		
Partner works on coffee^b^	102 (92.7%)	
On his own land	93 (91.2%)	
On family land	7 (6.9%)	
For others	7 (6.9%)	
Number of sacks of coffee (57 kg) produced last year	5 (0–40)	
Last year’s income from coffee (MXN)^c^	11,750 (0–120,000)	
Equivalent in USD PPP^d^ per family, per day	3.7	
House ownership		
Her own home	15 (10.6%)	
Partner	52 (37.0%)	
Both	19 (13.0%)	
In-laws	20 (14.2%)	
Father	17 (12.1%)	
Other	18 (13.0%)	
Women’s access to financial resources		
Own land	17 (12.1%)	
Small business	30 (21.3%)	
Cash availability	31 (22.0%)	
None	73 (51.8%)	
Social programs and support		
*Prospera*	98 (69.5%)	
Other government programs (Procampo, CONAFOR)	62 (44.0%)	
Remittances	24 (17%)	
International	17 (12.1%)	
National	7 (5.0%)	
Food insecurity^e^	38 (27.0%)	
Food insecurity severity	At least once a month	Almost every day
** Worried that there was not enough food for herself and her family**	18 (12.8%)	24 (17.0%)
** Bought food but was not enough and did not have money to buy more**	12 (8.5%)	7 (5.0%)
** Someone in the family had to skip meals or eat less because there was not enough food**	8 (5.7%)	7 (5.0%)

^a^*n* = 128.

^b^*n* = 111.

^c^*n* = 102.

^d^ PPP = Purchasing Power Parity, OECD 2017 published rates.

^e^ Two out of three of the statements below were positive during the past 12 months.

### Prevalence of intimate partner violence

Lifetime prevalence of IPV among ever partnered women was 66.4% (95% CI: 57.5–74.5%) and last 12 month prevalence was 24.2% (95% CI: 17.1–32.3%). As shown in Table 2, lifetime prevalence of partner physical violence was 64.8% (95% CI: 55.9–73.1%) and partner sexual violence was 25.8% (95% CI: 18.534.3%) among ever partnered women.

**Table 2:** Lifetime and last-12-month prevalence of IPV types.

	Lifetime prevalence among ever partnered	Last 12-month prevalence among ever partnered
	(95% CI) (*n* = 128)	(95% CI) (*n* = 128)
**Physical IPV**	64.8% (55.9–73.1%)	21.8% (15.1–30.0%)
**Sexual IPV**	25.8% (18.5–34.3%)	6.3% (2.7–11.9%)
**Physical and /or Sexual IPV**	66.4% (57.5–74.5%)	24.2% (17.1–32.3%)
**Considering partner controlling behaviors**		
**MC-IPV**	25.8% (18.5–34.3%)	n.d.^a^
**HC-IPV**	40.6 (32.0–49.7%)	n.d.^a^

^*a*^*n*.*d*.: no data, data on one-year occurrence of partner CB were not recorded.

There was a high prevalence of CB, with one in three (95% CI: 23.4–40.0%) ever partnered women having experienced more than four CB from their partners, and 35.2% (95% CI: 26.9–44.8%) having experienced between one and four. Interestingly, 17% of ever partnered women in the sample had experienced some form of control, but no physical or sexual IPV. Among these women, most reported being constantly humiliated or accused of being unfaithful, one woman reported high control from her partner and no physical or sexual partner violence. The prevalence of IPV with low-moderate CB was found to be 25.8% (95% CI: 18.5–34.3%), while it was 40.6% (95% CI: 32.0–49.7%) for IPV with high CB.

### Factors associated with IPV

As illustrated in Table 3, we found no statistical association between age or age at first cohabitation and IPV. Women who finished elementary school had a 0.63 times lower risk (95% CI: 0.4–0.9) of suffering physical lifetime IPV than those with lower levels of schooling, but no association was found with sexual IPV. Spending more than 12 hours a day in housework (highest tercile) increased the risk of IPV by 1.3 times (95% CI: 1.0–1.6); specifically, the risk physical IPV by 1.5 times (95% CI: 1.1–2.1), and the risk of sexual IPV by 1.7 times (95% CI: 1.0–3.1). Interestingly, a negative association was found between partner coffee-land ownership and experiencing sexual violence (RR = 0.45, 95% CI: 0.26–0.79; *p* = .007) and a tendency towards significance for the association between partner coffee-land ownership and physical IPV (RR = 0.78, 95% CI: 0.61–1.00; *p* = .074).

**Table 3:** Association of demographic, economic and partner alcohol abuse characteristics of participants with types of IPV and depression (*n* = 128).

	IPV (*n* = 85)		MC-IPV (*n* = 33)		HC-IPV (*n* = 52)		Depression (*n* = 23)	
	RR (95% CI)	*P* value	RR (95% CI)	*P* value	RR (95% CI)	*P* value	RR (95% CI)	*P* value
**Sociodemographic characteristics**								
**Age** ^***a***^		.100		.273		.097		.280
Age at first cohabitation^***a***^		.405		.793		.151		.855
Age at first cohabitation < 16	1.10 (0.86–1.41)	.461	0.84 (0.44–1.59)	.576	1.31 (0.92–1.87)	.151	1.30 (0.55–3.02)	.564
**Civil status**								
Married/ cohabitating	0.76 (0.59–0.98)	.115	1.09 (0.36–3.30)	.000	0.61 (0.44–0.84)	.027	0.64 (0.29–1.42)	.285
Separated	1.31 (0.94–1.82)	.251	n.c.^c^		1.64 (1.14–2.36)	.123	1.82 (0.53–6.27)	.320
Widowed	1.26 (0.92–1.72)	.333	1.16 (0.42–3.21)	.000	1.49 (0.99–2.22**)**	.177	0.54 (0.08–3.62)	.692
**Children**								
Five children or more	1.40 (1.11–1.75)	.009	1.61 (0.98–2.64)	.083	1.67 (1.19–2.33)	.006	1.32 (0.62–2.79)	.473
Death of offspring	1.48 (1.21–1.81)	.007	2.0 (1.26–3.18)	.035	1.78 (1.32–2.40)	.010	2.02 (0.96–4.25)	.066
**Education**								
Illiterate	---		---		---		---	
Incomplete elementary school	0.85 (0.65–1.13)	.280	0.88 (0.47–1.64)	.681	0.75 (0.49–1.15)	.181	1.9 (0.38–9.86)	.414
Elementary school	0.63 (0.43–0.93)	.013	0.56 (0.26–1.19)	.133	0.50 (0.28–0.88)	.009	2.50 (0.52–12.00)	.230
Middle or high school	0.92 (0.68–1.25)	.590	0.90 (0.49–1.65)	.719	0.89 (0.57–1.39)	.590	4.36 (1.05–18.16)	.018
**Ocupation**								
Hours spent in housework^*a*^		.087		.067		.214		.666
>12 hours spent in housework	1.29 (1.02–1.63)	.045	1.59 (0.98–2.61)	.075	1.40 (0.98–1.99)	.076	1.37 (0.64–2.93)	.417
Remunerated work	1.36 (1.09–1.70)	.025	1.77 (1.09–2.86)	.043	1.52 (1.08–2.14)	.041	0.78 (0.29–2.12)	.619
**Economic characteristics**								
*Prospera* ^*b*^	1.1 (0.82–1.46)	.517	1.06 (0.60–1.86)	.834	1.19 (0.77–1.85)	.416	0.57 (0.27–1.20)	.139
Partner grows coffee in his own land	0.77 (0.61–0.97)	.046	0.93 (0.48–1.78)	.823	0.60 (0.43–0.84)	.006	0.67 (0.32–1.42)	.300
Income from coffee ^*a*^ (hundreds of mxn)		.912		.758		.893		.733
Cash availability	1.02 (0.74–1.35)	.974	0.90 (0.46–1.79)	.765	1.06 (0.69–1.61)	.802	1.55 (0.70–3.43)	.285
One-year food insecurity	1.19 (0.93–1.52)	.198	1.30 (0.77–2.22)	.350	1.29 (0.90–1.85)	.201	0.96 (0.41–2.24)	.919
**Partner alcohol abuse**								
None	---		---		---		---	
Low	2.13 (1.29–3.49)	.001	2.94 (1.36–6.35)	.002	2.5 (1.12–5. 60)	.016	1.19 (0.43–3.29)	.735
Moderate	2.45 (1.49–4.06)	.000	2.78 (1.10–7.00)	.038	3.8 (1.78–8.2)	.000	1.86 (0.63–5.44)	.259
High	2.86 (1.79–4.59)	.000	3.33 (1.14–9.75)	.072	4.75 (2.31–9.78)	.000	1.90 (0.63–5.44)	.259

IPV: physical and sexual intimate partner violence; MC: moderate partner control, HC: high partner control.

^*a*^ Continuous variables. Calculated with Wilcoxon rank sum test.

^*b*^*Prospera* is a governmental conditional cash transfer program that provides women with some cash conditioned on the fulfillment of certain health related obligations and sending children to school.

^*c*^*n*.*c*.: not computed due to empty cells.

Together, lifetime physical and/or sexual IPV was shown to be positively associated with having five children or more, having had at least one child die, having remunerated work, spending more than 12 hours a day in housework, and partner alcohol abuse. In contrast, it was negatively associated with partner coffee-land ownership and having completed elementary school. Women in relationships in which their partner exerted high CB had 2.05-fold higher risk of having experienced physical violence (95% CI: 1.65–2.53), and 6.9-fold higher risk of having experienced sexual violence from their intimate partner (95% CI: 3.4–13.9), compared with women who reported not experiencing high CB. Seventy-three percent of women who reported low-moderate CB had experienced physical or sexual IPV, as well as 100% of those who reported high CB, in contrast to 28% of those who reported no CB.

### Differences in associated socioeconomic factors between MC-IPV and HC-IPV

In subgroup analysis HC-IPV retained the same associations except for hours spent in housework (continuous variable) and spending more than 12 hours on housework (dichotomic variable) which was marginally significant. In addition, a trend towards significance was found for the positive association between age and HC-IPV. Being married was found to be negatively associated with HC-IPV and a trend towards significance is noted for younger age at first cohabitation with a partner and HC-IPV. In contrast, MC-IPV retained a marginally significant association with hours spent in housework and spending more than 12 hours in housework, and a significant association between death of offspring, remunerated work, and low or moderate partner alcohol-abuse (Table 3).

**Fig 1:**
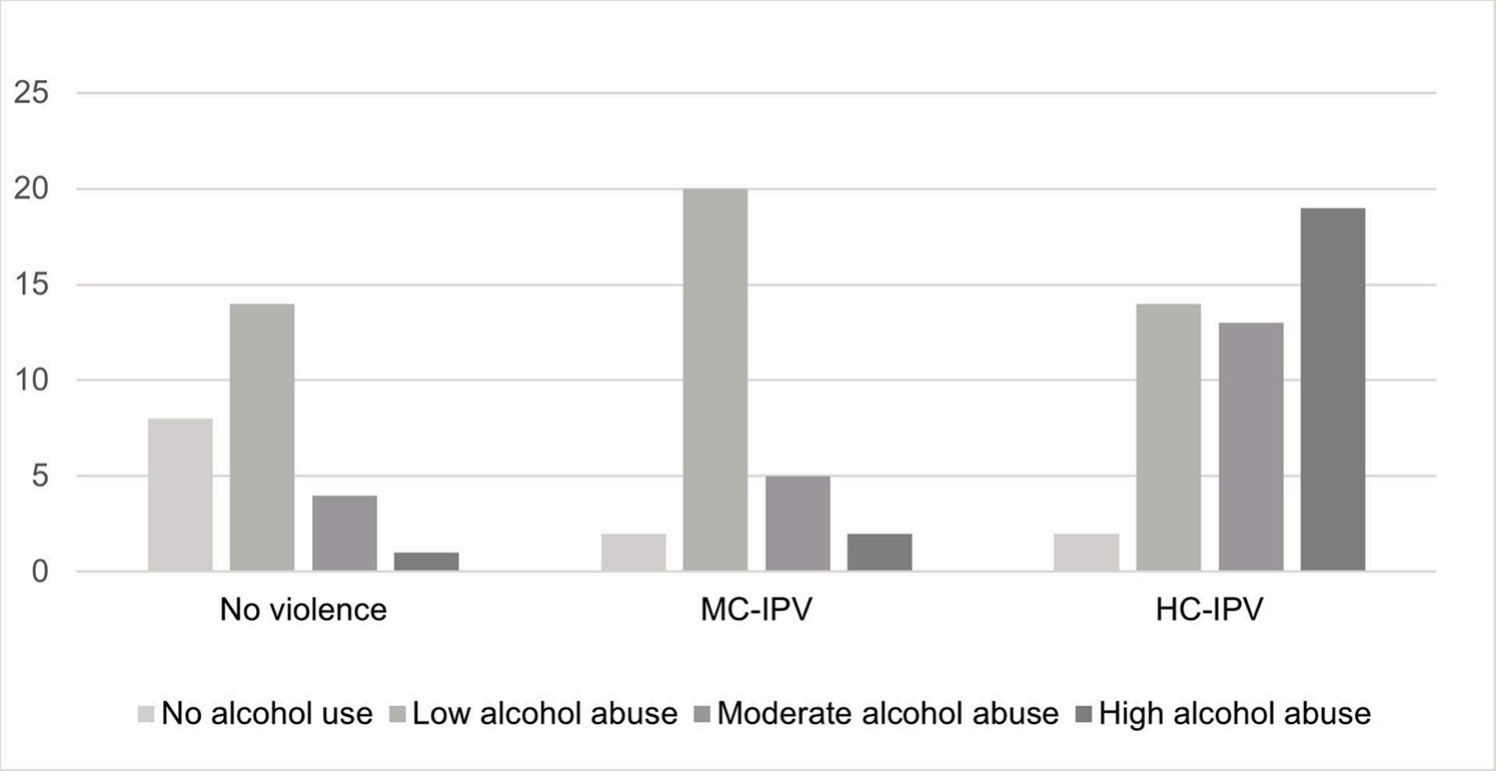
Partner alcohol abuse level and types of IPV. Fig 1 illustrates different patterns of partner-alcohol abuse by types of IPV considering CB.

Table 3 reveals a positive association between IPV and partner alcohol abuse. The odds of suffering IPV increased 3.06 times for each increase in the level of alcohol abuse compared to women whose partners did not abuse alcohol. Notwithstanding, 30.9% of women mentioned that their partners were not drunk when physical violence occurred and 29.6% mentioned they were drunk some, but not all the times. Interestingly, partners who perpetrate MC-IPV engage in moderate alcohol abuse more frequently, while partners who perpetrate HC-IPV engage more frequently in high alcohol abuse, as reported by women (see Fig 1). The differences in patterns of partner alcohol abuse between MC-IPV and HC-IPV were statistically significant (*p* = .001). Likewise, among women who reported high alcohol abuse from their partners, 68.18% also reported high CB in contrast to 23% among those whose partners did not engage in high alcohol abuse (*p* = .000).

**Fig 2:**
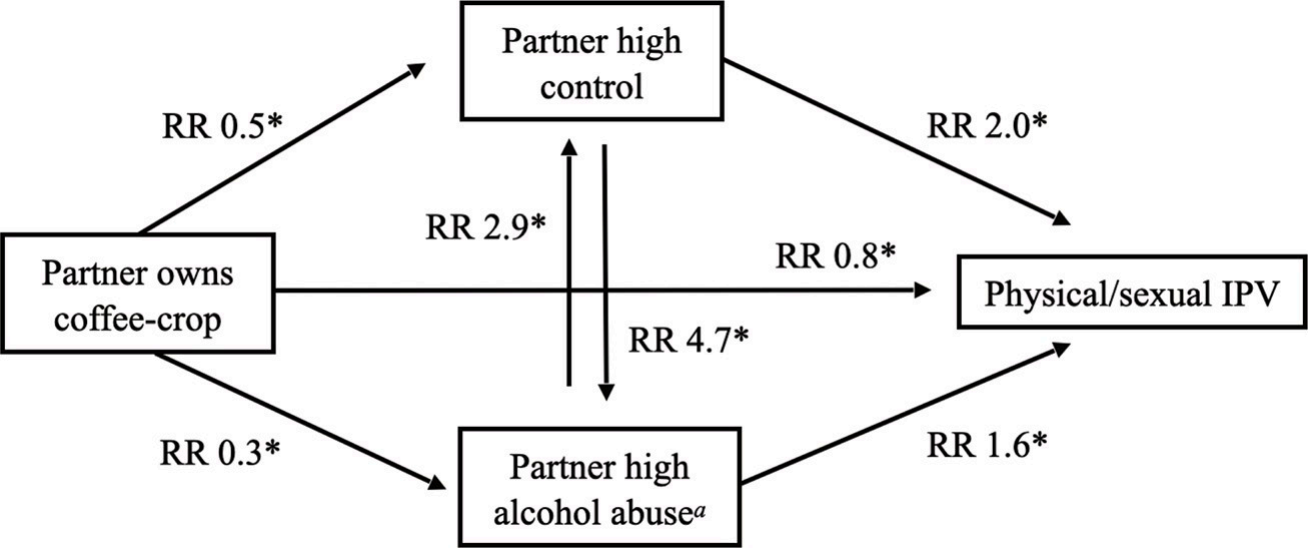
Associations between partner coffee-crop ownership, high control, high alcohol abuse and IPV. Fig 2 illustrates the bivariate associations between partner coffee crop ownership, high control, alcohol abuse and IPV. ^a^ High alcohol abuse was compared to none, low and moderate alcohol abuse, * *p* < .050.

Partner coffee-crop ownership was associated with a lower risk of IPV by 0.77 times (95% CI: 0.61–0.97; *p* = .046). In subgroup analysis it was found to be associated with a lower risk of HC-IPV by 0.60 times (95% CI: 0.43–0.84; *p* = .006), but not of MC-IPV. At the same time, partner coffee-crop ownership was found to be associated with a lower risk of partner high CB by 0.5 times (*p* = .003) and of partner high alcohol abuse by 0.3 (*p* = 0.001). When adjusting for CB and partner alcohol abuse, the association between coffee-crop ownership and IPV lost significance. Therefore, the relationship between coffee-crop ownership and IPV might be a result of a reduced risk of partner alcohol abuse and partner CB (Fig 2). In contrast, the significance of the association between spending more than 12 hours in housework and IPV was retained after adjusting for alcohol abuse and CB.

### IPV and mental health

Table 4 summarizes the findings of the associations between IPV subgroups and depression and anxiety symptoms. The prevalence for moderate-severe depression or anxiety symptoms was 16.3% (95% CI: 10.6–23.4%) and 8.5% (95% CI: 4.5–14.4%), respectively; overall, the prevalence of any of these (CMD) was 18.4% (95% CI: 12.4–25.8%). Among women with CMD, 73% had suffered IPV. When analyzing the relationship between depression and anxiety symptoms and the typology of violence considering partner control, HC-IPV held a significant association with moderate-severe depression symptoms (RR 5.8, 95% CI: 1.29–24.10; *p* = .004) and a trend towards significance with moderate-severe anxiety symptoms, but MC-IPV did not. Moreover, the risk of CMD was higher for HC-IPV than for severe IPV without considering the level of partner control (severe IPV: RR 4.6; 95% CI: 1.9–10.9 vs. HC-IPV: RR 6.1 (95% CI: 1.9–19.6). In logistic regression analysis including IPV severity, high control and CMD, the odds of suffering CMD among women who had suffered severe IPV decreased from 6.45 (95%CI: 2.31–18.02) to 4.82 (95% CI: 1.35–17.18) after adjusting for high control, suggesting that high control is partially driving the association between severe IPV and CMD.

**Table 4:** Association of types of intimate partner violence with symptoms of CMD (*n* = 128).

**Depression symptoms**							
	**No**	**Mild**			**Moderate-severe**		
	**%**	**%**	**RR (CI)**	***P* value**	**%**	**RR (CI)**	***P* value**
No IPV	69.77	25.58	---	---	4.65	---	---
Physical and/or sexual IPV	60.00	20.00	0.93 (0.49–1.79)	.832	20.00	4.3 (1.04–17.8)	.033
Physical IPV	61.45	20.48	0.93 (0.49–1.79)	.832	18.07	2.0 (0.72–5.76)	.200
Sexual IPV	36.36	30.30	2.2 (1.19–4.10)	.018	33.33	3.96 (1.74–8.99)	.001
MC-IPV	69.70	21.21	0.87 (0.38–1.98)	.738	9.09	1.95 (0.35–11.03)	.439
HC-IPV	53.85	19.23	0.98 (0.47–2.0)	.959	26.93	5.8 (1.29–24.10)	.004
**Anxiety symptoms**							
	**No**	**Mild**			**Moderate-severe**		
	**%**	**%**	**RR (CI)**	***P* value**	**%**	**RR (CI)**	***P* value**
No IPV	86.05	11.63	---		2.33	---	
Physical and/or sexual IPV	69.41	18.82	1.62 (0.64–4.12)	.299	11.76	5.06 (0.67–38.24)	.098
Physical IPV	71.08	18.07	1.36 (0.57–3.25)	.489	10.84	2.44 (0.55–10.81)	.218
Sexual IPV	60.61	24.24	1.77 (0.81–3.89)	.158	15.15	2.40 (0.78–7.34)	.119
MC-IPV	78.79	12.12	1.04 (0.30–3.38)	.100	9.09	3.90 (0.43–35.89)	.311
HC-IPV	63.46	23.08	1.95 (0.83–4.57)	.147	13.46	5.78 (0.74–45.2)	.067
**CMD symptoms**							
	**No**	**Mild**			**Moderate-severe**		
	**%**	**%**	**RR (CI)**	***P* value**	**%**	**RR (CI)**	***P* value**
No IPV	65.12	31.71	---		4.65	---	
Physical and/or sexual IPV	49.41	35.38	1. 12 (0.64–1.95)	.697	23.53	5.06 (1.24–20.65)	.007
Physical IPV	50.60	35.38	1.12 (0.64–1.95)	.697	21.69	2.44 (0.88–6.77)	.087
Sexual IPV	36.36	45.45	1.47 (0.84–2.57)	.201	33.33	2.88 (1.38–6.01)	.004
MC-IPV	63.64	27.59	0.87 (0.41–1.83)	.795	12.12	2.60 (0.51–13.38)	.394
HC-IPV	40.38	41.67	1.31 (0.73–2.38)	.365	30.77	6.62 (1.61–27.19)	.001
**Suicidality**							
	**No**	**Yes**					
	**%**	**%**	**RR (CI)**	***P* value**			
No IPV	83.33	16.67	**---**	**---**			
Physical and/or sexual IPV	75.29	24.71	1.48 (0.69–3.21)	.367			
Physical IPV	74.70	25.30	1.59 (0.73–3.44)	.266			
Sexual IPV	60.61	39.39	2.47 (1.32–4.63)	.005			
MC-IPV	90.91	9.09	0.55 (0.15–1.95)	.338			
HC-IPV	65.38	34.62	2.08 (0.96–4.50)	.050			

Besides depression and anxiety symptoms, suicidality showed a marginally significant association with HC-IPV 2.08 (95% CI: 0.96–4.50) (*p* = .050). Twenty-two percent (*n* = 31) of women in our sample had thought at least once in the two weeks prior to the survey that they would be better off dead than alive. Of those women, ten had thought about this almost every day, of which eight reported having experienced HC-IPV, one MC-IPV and one reported she had never experienced IPV. Moreover, all three women who had thought of a specific way to kill themselves reported having suffered HC-IPV.

## Discussion

This study examined the scope and characteristics of IPV in a rural community in Chiapas, and the association of IPV—considering partner control—with depression and/or anxiety symptoms. Our results show that the experience of physical and/or IPV is frequent, with 66.4% of ever partnered women having experienced it throughout their lifetimes, a prevalence much higher than the national and state estimates. While our study estimates a lifetime prevalence of 64.8% of physical and 25.84% of sexual IPV, national estimates in 2016 where 17.9% and 6.5%, and Chiapas’ estimates were 17% and 5.8%, respectively [45]. Our study supports existing literature that reports higher prevalence of IPV in settings of poverty [46, 47].

Our results might not be representative of rural Mexico. Still, many rural communities share structural and economic characteristics such as poverty, *ejidal* structure, almost exclusive male land-tenure, marginalization and lack of access to social and legal services. Consequently, we believe our findings shed light on an important gap of the ENDIREH, as it may underestimate the prevalence of IPV in rural and indigenous areas. The differences between our results and State estimates call for the use of survey methods that consider variation within states and municipalities such as Large-Country Lot Quality Assurance Sampling methods (LC-LQAS) [48]. LC-LQAS could avoid underestimation of IPV and help plan the distribution of mental health, social and legal services accordingly.

To the authors’ knowledge, this is the first study that examines the combination of severity of physical and sexual IPV considering partner control and the consequences for women’s mental health in rural Mexico. Previous studies have categorized IPV considering the type of violent acts as physical, sexual, economic and emotional/psychological, regarding the later as a less severe form of violence. However, these controlling behaviors exert a cumulative damage throughout the lifespan and are frequently experienced along with physical and sexual violence. In our study, all women who reported high CB, and most women who reported low-mild CB also reported having experienced physical and/or sexual violence from their partner. Previous studies have shown that, while SCV can be frequent and severe, violent acts in IT are usually more frequent and more severe [8].

Our findings are in concordance with Johnson’s classification of different typologies of IPV, given that our constructs MC-IPV (similar to SCV) and HC- IPV (similar to IT) have shown to be differently related to various socioeconomic factors, as well as to mental health outcomes. Of note, HC-IPV was positively associated with having 5 or more children and higher partner alcohol abuse, with a trend towards significance for younger age and first union, and negatively associated with partner coffe-crop ownership and elementary school completion—which could be considered proxies for traditional gender beliefs and norms [2, 37]. Notwithstanding, in contrast with Johnson’s findings [2], HC-IPV was more frequent than MC-IPV in our study setting. This difference highlights the importance of context when studying IPV, including structural gender inequality and traditional gender beliefs and norms. Likewise, it raises questions about the extent of the impact that poverty has in shaping interpersonal relationships.

Interestingly, the conditional cash transfer program (*Prospera)* was not associated with lower risk of IPV as suggested previously [49, 50]. This finding is possibly related to the fact that: a) the cash transfer does not translate in cash availability nor economic independence for women in this community, and/or b) the program does not transform noxious gender norms that support partner CB but in fact makes use of them for programmatic purposes [51]. The latter is of special relevance in a setting where HC-IPV is the dynamic of IPV experienced more frequently. Of note, Bobonis and colleagues’ results [50] show that while the CCT program is associated with a reduced risk of physical IPV, it is likewise associated with an increased risk of emotional IPV, considered CB in the present study.

Among the economic characteristics, only partner coffee-crop ownership was associated with IPV, and specifically with a reduced risk of HC-IPV. After adjusting for high alcohol abuse and CB this association resulted not to be statistically significant, suggesting that these could be confounders in the association between coffee-crop ownership and lower risk of IPV. Interestingly, a clear association was found between not working exclusively on his own coffee crop and both, high alcohol abuse and high CB. A possible explanation for this association is the relationship of coffee-crop ownership with social status within the community. In this setting, owning a coffee-crop secures a seat at the community assembly and increases men’s social networks, status and political leverage within the community. Having to work on someone else’s coffee crop is usually related to not having enough land or not having it at all, which entails relying economically on others or temporary jobs and significant economic distress. Sociological theories suggest that men who hold a lower social status may be more likely to engage in heavy drinking, behave aggressively, and exert CB and violence towards women, possibly reflecting the few resources available for them to secure some power and control in the context of a “masculinity crisis” [52–56].

Likewise, the fact the high partner control is positively associated with high alcohol abuse point towards a common origin, possibly rooted in gender beliefs and norms [16, 57]. Our findings show that alcohol use is not necessary, nor enough for IPV to happen: 68% of women reported that their partner was not drunk all the times when violence happened, and one third of participants reported their partner was never drunk when violence occurred. Despite conflicting evidence on the role of alcohol abuse in IPV [17], in our study setting being drunk was indeed regarded as a “time-out” behavior in which a man “got crazy”, “was not himself”, and therefore could not be held accountable for his actions [16, 58]. Women who participated in the survey had to be specifically asked about IPV acts while her partner was drunk. Many times, women who had answered negatively (disregarding his actions while drunk) changed to a positive answer after being asked whether her partner had ever exerted violence when he had been drinking.

Besides alcohol abuse, we found strong associations between mental disorders and IPV similar to those in previous studies [1, 37, 59]. The strong association of sexual violence with depression and suicidality shown in this study, is well known for mental health professionals. The higher risk of sexual IPV in contexts with traditional gender norms and roles, partner alcohol abuse, and jealousy and control has also been published [60]. The fact that CB increased almost 7-fold the risk of sexual IPV calls for higher attention to CB in IPV dynamics to reduce the risk of sexual IPV and negative mental health outcomes. We contribute to the literature the different effects of MC-IPV and HC-IPV on women’s mental health in rural Mexico, with HC-IPV having the most impact on increasing the risk of moderate-severe depression, anxiety and suicidality. This association between HC-IPV and psychiatric symptoms is in concordance with previous literature on IT and SCV [6–8], and on the effects of long-term terror and captivity in woman’s mental health [1, 61]. Our study showed that HC-IPV is increases over five times the risk of moderate or severe depression, two times the risk of suicidality and a trend towards significance was noted for an increase of over five times the risk for moderate or severe anxiety. These results are higher that previous studies which have found a 2.5 higher risk for depression [7]. This difference could be due to the adverse context of poverty and marginalization which also increase the risk for other traumatic and adverse experiences [62]. Cumulative trauma throughout the lifetime has been shown to have a higher negative impact on mental health that isolated traumatic experiences, especially when trauma is interpersonal [63]. In addition, partner CB, as well as poverty, add an element of captivity to the experience of physical and sexual violence. The effects of long-term captivity and chronic trauma on mental health have been also described in victims of the holocaust, war prisoners and war veterans [61]. Considering CB a lesser kind of violence in public health and clinical practice disregard the evidence pointing towards the important impact these behaviors have on women’s mental health. American psychiatrist Judith Herman called, since 1992, for the inclusion of “Complex Post-Traumatic Stress Syndrome” (C-PTSD) as a nosologic entity to account for the effects of violence in the context of captivity and control [13], which entails posttraumatic stress symptoms as well as alterations in emotional regulation, interpersonal relationships and self-perception. This diagnostic category was recently added to the 11^th^ revision of the International Classification of Diseases (ICD-11) [64]. Given the high prevalence of HC-IPV found in this study setting, further research should explore the prevalence of C-PTSD in rural or marginalized regions in Mexico.

Our small study sample did not support the analysis of more complex multivariate models of demographic and socioeconomic factors, still, our results are similar to wider cross-cultural studies that show a significant association between, school attainment and number of children [46, 65]. Similarly, the small sample size could result in a lack of power to detect some associations between risks and outcomes, especially in those cases in which a trend towards significance is noted (Tables 3 and 4). Likewise, the study design does not allow us to evaluate temporality between risks and outcomes. Notwithstanding, people in this community have lived in poverty since the first families settled there, suggesting that temporality between most socioeconomic factors and IPV can be established.

### Implications for global mental health practice

The pathway through which IPV influences mental health is complex and context specific. This complexity must be considered in the design of IPV and mental healthcare services to adequately assist impoverished women in rural communities. Women who suffer from CB from their partners could be at higher risk of both CMD and physical or sexual IPV. Health care providers could ask about partner CB to capture women at risk of severe IPV, mental health disorders, and even feminicide, given that women experiencing high levels of partner control are at a higher risk of being killed by their partners [66]. Simultaneously, mental health services must be accessible for marginalized women, who bear the highest burden of social determinants for mental illness.

If healthcare is to serve the purpose of human development, we must strive to move away from basic healthcare packages for the poor and push for comprehensive physical and mental healthcare that is accessible from the first level of care [67, 68]. Mental health programs that focus exclusively on prevention and do not consider structural gender inequality, inequitable gender beliefs and norms, and its role in supporting CB and IPV may fail to deliver care for women who have suffered severe IPV and other experiences of gender-based violence. Consequently, providers in settings of poverty who are not equipped to respond to disclosure of IPV or other forms of trauma, and who work in siloed programs with little or no linkage to specialized services, may soon become frustrated or burn-out as has been shown to happen when resources for care are scarce [69, 70].

Effective models of mental healthcare at the community level must be integrated. We advocate for considering the complexity of the experiences of women, men, boys and girls in impoverished settings when designing and implementing these models. Such patient-centered care must include a budgetary allocation that correspond to the burden of disease (to assure human capacity and a stable medication supply-chain), as well as an adequate distribution of human resources [33] which can range from community health workers to specialists, organized in stepped models of care [71–75].
